# Improving border irrigation performance with predesigned varied-discharge

**DOI:** 10.1371/journal.pone.0232751

**Published:** 2020-05-06

**Authors:** Kaihua Liu, Xiyun Jiao, Weihua Guo, Yunhao An, Mohamed Khaled Salahou

**Affiliations:** 1 State Key Laboratory of Hydrology–Water Resources and Hydraulic Engineering, Hohai University, Nanjing, China; 2 College of Agricultural Engineering, Hohai University, Nanjing, China; 3 Cooperative Innovation Center for Water Safety and Hydro Science, Hohai University, Nanjing, China; Hellenic Agricultural Organization - Demeter, GREECE

## Abstract

Insufficient water resources restrict wheat production in the North China Plain, so it is urgent and essential to improve the border irrigation performance and water use efficiency. This study developed a predesigned varied-discharge irrigation scheme in the closed-ended border. Field treatments, including continuous-discharge (CD), increased-discharge (ID) and decreased-discharge (DD) border irrigation tests, were conducted to evaluate the irrigation performance of the proposed varied-discharge scheme. The DD border irrigation treatment had great application efficiency (AE), distribution uniformity (DU) and requirement efficiency (RE), and its comprehensive evaluation indicator (Y) was also significantly higher than other treatments. DD treatment achieved the average AE, DU, RE and Y values of 91.4%, 95.5%, 99.5% and 95.4%, respectively. Furthermore, the hydraulic simulation model WinSRFR was used to optimize the scheme of predesigned varied-discharge border irrigation, and sensitivity analyses of infiltration parameters, roughness coefficient, slope and inflow rate were carried out. The results indicate that the predesigned varied-discharge border irrigation scheme can improve the irrigation performance, and the DD border irrigation scheme has more satisfactory robustness than that of the ID border irrigation scheme.

## Introduction

The North China Plain is one of the most important agricultural regions in China [1]. As rainfall is insufficient and uneven (mainly concentrated in summer), irrigation water for wheat is mostly pumped from groundwater, and irrigation has been identified as one of the main factors of groundwater drawdown [2–5]. Border irrigation is the most widely used irrigation method in the North China Plain [6]. It is urgent and essential to increase the border irrigation water use efficiency and avoid further overexploitation of groundwater.

In the border irrigation system, the variable measures are soil infiltration properties, roughness coefficient, border dimensions (length and width), slope, inflow rate and cut-off time (or cut-off distance) [7,8]. Although the soil infiltration properties and roughness coefficient have been proven to affect the performance of border irrigation [9–11], they are difficult to control artificially, so the soil infiltration properties and roughness coefficient are regarded as input parameters rather than controlled variables in border irrigation design. Optimizing the border dimensions and slope can improve the irrigation performance [12,13]. The border dimensions and slope are designed before planting according to the topography, which are difficult to change afterwards. Therefore, the border dimensions and slope are mostly regarded as fixed input parameters rather than control variables when irrigating at different growth stages. At present, the optimal design for inflow rate (including cut-off) is a simple and commonly used way to improve border irrigation performance [14].

According to whether the inflow is adjusted in the irrigation process of a border, border irrigation is divided into continuous-discharge (CD) and varied-discharge irrigation. CD irrigation is a traditional irrigation method with a constant inflow rate across the entire border, and many researches have been conducted to improve CD irrigation. On the basis of analyzing the influence of inflow and cut-off time on distribution uniformity, Santos [15] proposed the optimum combination of inflow rate and cut-off time. Bai et al. [16] improved the irrigation performance by optimizing the cut-off time. Salahou et al. [6] proposed a reasonable inflow rate and distance-based cutoff to improve border irrigation performance and water use efficiency in the closed-ended border irrigation system. Although these studies improved the irrigation performance of CD irrigation to a certain extent, CD irrigation still has unsatisfactory application efficiency and distribution uniformity [17,18].

Varied-discharge irrigation is described by increasing or decreasing the inflow rate before the water advance phase is completed. Compared with the continuous-discharge irrigation, the varied-discharge irrigation further improves the irrigation performance. The varied-discharge irrigation usually includes real-time control irrigation, surge irrigation and predesigned varied-discharge irrigation. The real-time control system for surface irrigation has been extensively studied due to its good irrigation performance and reduced labor requirements [19–21]. The system uses a lot of sensors to monitor the process of surface flow advance, calculates soil infiltration properties in real time, and simulates the irrigation to determine the best inflow rate (or cut-off time). The disadvantage of this irrigation system is that it needs a lot of sensors and complex calculations in a short time. Economically and technically, the real-time control system is difficult to be widely used in the North China Plain. Surge irrigation is a special irrigation method for the intermittent application of water to furrows or borders in a series of periodic on and off periods of specific time spans [18]. Properly managed, and under long field conditions, surge irrigation can lessen deep percolation and improve irrigation performance [22–24]. However, the adaptability study of surge irrigation demonstrated that surge irrigation offers no advantage over conventional continuous-flow border irrigation if the border length is less than 100 meters in the North China Plain [25]. In China, agricultural practice is mainly carried out on small farms [13], and the border length is generally not long. Furthermore, to increase the border irrigation water use efficiency and yield, the border length is usually no more than 100 meters [26]. In this case, surge irrigation will not significantly improve the irrigation performance in the North China Plain.

The predesigned varied-discharge irrigation, including increased-discharge (ID) and decreased-discharge (DD) border irrigation, is simple and feasible. Many studies have indicated that satisfactory irrigation performance can be obtained by reducing the initial inflow after the advance water reaches the furrow end [27,28]. Valipour [29] used SIRMOD software to simulate the cutback irrigation method in open-ended border irrigation systems, and indicated that the cutback irrigation method can increase irrigation efficiency by 11.66%. Vázquez-Fernández et al. [30] compared the distribution uniformities between continuous-discharge and ID (the discharge doubled when the water reached a quarter or half of the furrow’s length) furrow irrigations, and concluded that ID irrigation was an efficient irrigation in closed-ended furrows.

Previous studies only focused on the problem of predesigned varied discharge in furrow irrigation or open-ended border irrigation systems. However, for crops in the North China Plain, especially winter wheat, the main crop, closed-ended border irrigation is the main irrigation method [6]. Additionally, previous studies change the discharge in a special proportion, mostly doubling or halving, without finding the optimal numerical varied-discharge scheme. Hence, the objectives of this study were as follows: (1) to preliminarily evaluate the irrigation performance of CD, ID and DD border irrigation methods through field tests; (2) to determine the optimal predesigned varied-discharge border irrigation scheme with the aid of the hydraulic simulation model WinSRFR; (3) to assess the sensitivity of the soil infiltration properties, roughness coefficient and slope on the irrigation performance when the optimal predesigned varied-discharge border irrigation scheme is used; and (4) to provide recommendations for improving the border irrigation performance and water use efficiency in the North China Plain.

## Materials and methods

### Study area

**Fig 1:**
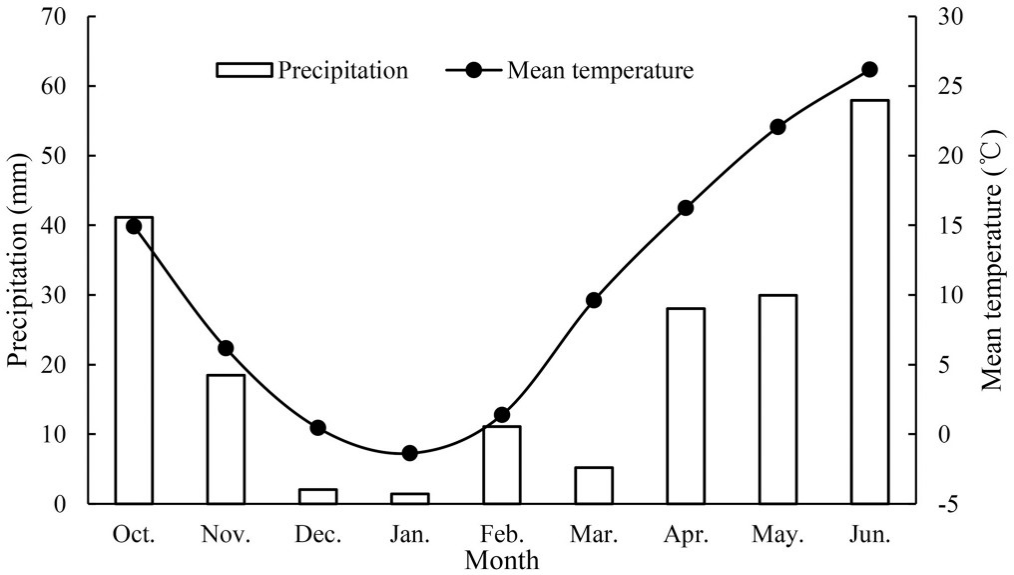
Average monthly precipitation and temperature from 2013 to 2018 during winter wheat growing seasons.

The experimental filed is located at the Nanpi Ecological Agricultural Experiment Station of Chinese Academy of Sciences, Hebei Province, China (116°40 ′ E, 38°06 ′N). This area has a continental monsoon climate. Winter wheat is the main crop to be cultivated, usually planted in October and harvested in June next year. The mean annual precipitation at the study site is 567.4 mm, with approximately 73%, 13%, 11%, and 3% of the annual precipitation occurring during the summer, autumn, spring and winter, respectively [6]. The mean annual temperature, sunshine hours and radiation are 12.3 °C, 2938.6 hr and 5592.3 MJ cm^-2^, respectively. And the average annual rainfall during the winter wheat growing season from 1996 to 2016 was 125 mm [31]. The average monthly precipitation and temperature from 2013 to 2018 are shown in Fig 1. Weather data were obtained from the Botou Weather Station of China Meteorological Data Service Center, which was about 14 kilometers away from the study site. The experimental field is high in the south and low in the north, with an overall slope of about 0.0025. Closed-ended border irrigation is the main irrigation method, and irrigation water mostly comes from groundwater. The soil particle size was classified as a silt loam (63.12% silt, 29.79% sand, and 7.09% clay on average). The average dry bulk density is 1.49 g/cm^3^ at a depth of 1 m (1.40 g/cm^3^ at 0–20 cm, 1.49 g/cm^3^ at 20–40 cm, 1.56 g/cm^3^ at 40–60 cm, 1.51 g/cm^3^ at 60–80 cm, and 1.48 g/cm^3^ at 80–100 cm).

### Experimental design and data measurement

Experiments were conducted in the jointing stage, which is the most important irrigation period in the wheat growing process in the North China Plain [32–34]. Three schemes of CD, ID and DD border irrigations were carried out. Like the borders used by local farmers, all the test borders were 0.0025 in slope, 80 m in length, 3.7 m in width and closed-ended. The border slope of 0.0025 and border length of 80m are favorable for higher water use efficiency and yield [26,35]. The working width of the local seeder is 1.8m. And the border width of 3.7 m is slightly larger than twice the working width of the seeder, which is convenient for the operation of the seeder. The required application water depth was 60 mm [6,35]. The inflow rate is limited by water supplies, soil texture and other factors, and too large or too small of an inflow rate is not feasible. The general inflow rate ranges approximately from 3 to 7 L s^-1^m^-1^ for border irrigation in the North China Plain [6,35]. Hence the CD experimental inflow rates were divided into three levels (high, moderate, and low, at 7 L s^-1^m^-1^, 5 L s^-1^m^-1^, and 3 L s^-1^m^-1^, respectively), and the distance-based cut-off ratios were determined according to the inflow rate [6]. Based on the existing research [30] and irrigation experience, exploratory ID and DD experiments were designed. The advances of the inflow rate change were a quarter and half of the border length [30], and the water cut-off was determined according to the flow rate. As shown in Table 1, six treatments (CD1, CD2 CD3, ID1, ID2 and DD) were designed and each treatment was repeated in 2–3 borders (CD1, CD2, CD3 and ID2 was repeated in 2 borders, ID1 and DD was repeated in 3 borders). A total of 14 border irrigation experiments were carried out.

**Table 1:** Experimental treatment design of continuous-discharge irrigation and varied-discharge irrigation.

Treatment	Initial inflow rate (L s^-1^m^-1^)	First change		Second change		Cut-off distance ratio
		Advance of inflow rate change (m)	Inflow rate after change (L s^-1^m^-1^)	Advance of inflow rate change (m)	Inflow rate after change (L s^-1^m^-1^)	
CD1	3	—	—	—	—	0.90
CD2	5	—	—	—	—	0.85
CD3	7	—	—	—	—	0.80
ID1	3	20	5	40	7	0.80
ID2	3	20	4	40	5	0.85
DD	7	20	5	40	4	0.85

For each border, the relative elevations were measured every 5 m using the optical level DSZ2 (±1mm). Then linear least square regression is used for the relative elevation data of the same border, and the slope of the line is the border slope [36]. The soil moistures were measured before and after irrigation by the gravimetric method. Then the *a* and *k* parameters of the Kostiakov equation can be calculated using the volume balance method [35]. The roughness coefficient *n* is difficult to measure or calculate directly. In this study, *n* was obtained by the trial and error approach with the aid of the widely recognized hydraulic simulation model WinSRFR [6,37]. Specifically, a value was assigned to the *n* parameter and entered into the WinSRFR model, and the simulated advance and recession trajectories were compared with the observed data. If the fit was poor, a new value was assigned to the *n* parameter. This process was repeated until the simulated trajectories were in good agreement with the field data. The inflows of every border were measured by the electromagnetic flow meter (accuracy of ±1.5%).

### Irrigation performance analysis

The irrigation performance was usually evaluated by application efficiency (AE), distribution uniformity (DU) and requirement efficiency (RE) [6,16]. The AE is defined as the ratio of the average infiltrated water depth in the root zone and the average applied water depth. The DU is a measure of how uniformly water is applied across the border. The RE is defined as the ratio of the average infiltrated water depth in the root zone and the required water depth. AE, DU and RE reflect different aspects of irrigation performance. To better explain the irrigation performance, a comprehensive evaluation indicator Y was used for final evaluation [38]. Y has the maximum value of 100%, and the larger Y is, the better the irrigation performance is.

$$\text{AE}=\frac{\frac{1}{n}\sum_{i=1}^{n}h_{i}}{z_{A}}$$ (1)

$$\text{DU}=1-\frac{\frac{1}{n}\sum_{i=1}^{n}\left|z_{i}-z_{A}\right|}{z_{A}}$$ (2)

$$\text{RE}=\frac{\frac{1}{n}\sum_{i=1}^{n}h_{i}}{z_{r}}$$ (3)

$$\text{Y}=\sqrt[{3}]{\text{AE}\times \text{DU}\times \text{RE}}$$ (4)

where *h*_i_ is the infiltrated water depth in the root zone; *z*_i_ is the infiltrated water depth; *z*_*A*_ is the average infiltrated water depth and $z_{A}=\frac{1}{n}\sum_{i=1}^{n}z_{i}$; *n* is the number of stations along the border length; and *z*_r_ is the required water depth.

### Sensitivity analysis

Considering the spatiotemporal variability of the input parameters (infiltration parameters, roughness coefficient, slope and inflow rate), sensitivity analyses should be conducted to demonstrate the robustness of the proposed system [39,40]. There is a certain correlation between *k* and *a* [41], so these two parameters should be considered as a whole. The range of each parameter is determined based on the field data.

## Results and discussion

### Irrigation tests performance

Border irrigation parameters, obtained by averaging the parameters of the experimental borders of the same treatment, are shown in Table 2. Coefficients of variation (CV) of *k*, *a* and *n* were 2.4%, 1.1% and 1.7%, respectively, and the difference among the treatments was no more than 10%. The variability of *S* was a little larger (CV = 9.8%), but the differences of different treatments are mostly within 20%. These results indicate that no large variation in the border irrigation parameters distribution occurred within each treatment. These data were analyzed using a Duncan test to further determine differences between treatments, and the results also showed that there was no significant difference in irrigation parameters among the treatments.

**Table 2:** Observational data of border slope, Kostiakov parameters and roughness coefficient.

Treatment	Border slope *S*	Kostiakov parameters		Roughness coefficient *n*
		*k*(mm·min^-α^)	*α*	
CD1	0.0023a	6.679a	0.69a	0.098a
CD2	0.0022a	6.941a	0.70a	0.098a
CD3	0.0022a	7.037a	0.71a	0.095a
ID1	0.0024a	6.665a	0.69a	0.098a
ID2	0.0026a	6.731a	0.70a	0.100a
DD	0.0022a	6.641a	0.70a	0.095a

Within each column, different letters indicate significant differences at *p* = 0.05, based on the Duncan test.

**Fig 2:**
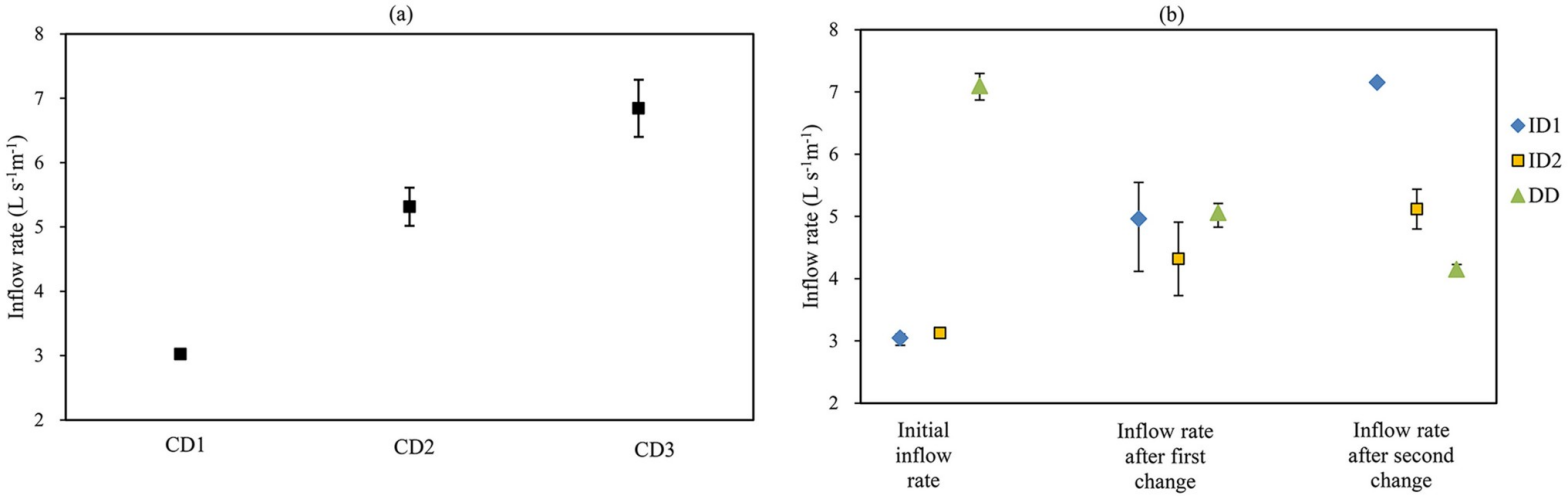
Measurement results of inflow rate for (a) continuous-discharge irrigation treatment, and (b) varied-discharge irrigation treatment. The vertical bars indicate the standard deviations of the inflow rate; for some points, the vertical bars are too small to be seen.

The measurement results of inflow rate are shown in Fig 2. In the border irrigation system, it is difficult to control the inflow rate accurately. And this difficulty will be more prominent when adjusting inflow rate during irrigation. But overall, the errors of inflow rate were almost within 10%.

The irrigation performance indexes (AE, DU, RE and Y) of each treatment are shown in Table 3. For all the treatments, the RE values were greater than 98%, which meant that the cut-off distance ratios were conservative, and the irrigation satisfied the required water depth in the root zone. For the CD treatments, the AE values ranged from 78.5% to 83.9% and the DU values ranged from 86.4% to 93.5%. Overall, the treatment for which the inflow rate was 5 L s^-1^m^-1^ and the cut-off distance ratio was 0.85 (CD2) was more satisfactory. The results were consistent with the closed-ended border irrigation testes conducted by Salahou et al. [6]. For the ID treatments, the ranges of the irrigation performance indexes were similar to those of the CD treatments, and there was no significant difference in irrigation performance between CD and ID treatment. The AE, DU and Y values of DD treatment were the largest, which were 91.4%, 95.5% and 95.4% respectively, and were significantly higher than those of CD and ID treatments. From the experiment data, the DD border irrigation is recommended.

**Table 3:** Irrigation performance of each treatment.

Treatment	AE (%)	DU (%)	RE (%)	Y (%)
CD1	78.5c	86.4de	99.6a	87.7c
CD2	82.8bc	93.5ab	100a	91.8b
CD3	83.9bc	88.2cd	100a	90.4b
ID1	80.0bc	82.6e	100a	87.1c
ID2	85.7ab	91.0bc	99.8a	91.9b
DD	91.4a	95.5a	99.5a	95.4a

Within each column, different letters indicate significant differences at *p* = 0.05, based on the Duncan test.

### Optimal border irrigation scheme simulation

There are too many varied-discharge border irrigation schemes to test each one. The experimental conclusion may not be all-inclusive. Hence, numerical simulation is needed to verify the test results and find the optimal scheme. To avoid the influence of different infiltration parameters, slopes and roughness coefficients, all the simulations were based on the same border irrigation parameters (*S* = 0.0025, *k* = 6.782, *a* = 0.7, *n* = 0.1). The advances of inflow rate change were a quarter and half of the border length (20 and 40 m) and the inflow rate step was 0.5 L s^-1^m^-1^, ranging from 3 to 7 L s^-1^m^-1^.

Through WinSRFR model simulation, appropriate schemes of ID and DD border irrigation were obtained. The top ten schemes of each group with the maximum Y values are shown in Table 4. The AE, DU, RE and Y values were 96.0%, 92.2%, 96.3% and 94.8%, respectively for the optimal ID scheme (ID01) and 97.1%, 94.9%, 97.9% and 96.6% respectively for the optimal DD scheme (DD01). The independent sample t-test (*p* = 0.05) indicated that the DU, RE and Y values of DD scheme were significantly higher than that of ID schemes. The AE, DU, RE and Y values of all 20 schemes were greater than 90%, and the mean values of the AE, DU and RE were greater than 94% and 95% for the ID and DD schemes, respectively. Although the irrigation performance of the DD border irrigation was better than that of the ID border irrigation, the ID and DD schemes were both quite satisfactory. In addition, satisfactory irrigation performance was achieved when the ranges of the initial inflow rate, the inflow rate after the first change and the inflow rate after the second change were almost 4–5 L s^-1^m^-1^, 5–5.5 L s^-1^m^-1^ and 6–7 L s^-1^m^-1^, respectively, for the ID border irrigation, and 6.5–7 L s^-1^m^-1^, 5.5–6.5 L s^-1^m^-1^ and 4–4.5 L s^-1^m^-1^, respectively, for the DD border irrigation. The appropriate cut-off distance ratio was closely related to the final inflow rate rather than the initial inflow rate or the inflow rate after the first change. This also means that the larger the final inflow rate is, the smaller the cut-off distance ratio.

**Table 4:** Top ten schemes of ID and DD border irrigation.

Scheme number	Initial inflow rate (L s^-1^m^-1^)	Inflow rate after first change (L s^-1^m^-1^)	Inflow rate after second change (L s^-1^m^-1^)	Cut-off distance ratio	AE (%)	DU (%)	RE (%)	Y (%)
ID01	5.0	5.5	7.0	0.70	96.0	92.2	96.3	94.8
ID02	4.5	5.5	6.0	0.75	94.0	92.2	98.1	94.8
ID03	4.0	5.0	7.0	0.75	95.3	92.3	96.8	94.8
ID04	4.5	5.0	6.0	0.70	93.5	92.7	98.2	94.7
ID05	4.0	4.5	5.5	0.75	93.9	92.6	97.6	94.7
ID06	4.0	5.5	6.0	0.75	93.3	91.9	98.4	94.5
ID07	4.0	5.0	6.0	0.75	92.9	92.4	98.3	94.5
ID08	4.0	4.5	7.0	0.70	94.2	92.1	97.2	94.5
ID09	3.5	5.5	6.0	0.75	93.1	91.9	98.3	94.4
ID10	4.5	6.0	7.0	0.70	95.8	91.7	95.8	94.4
DD01	6.5	6.0	4.5	0.80	97.1	94.9	97.9	96.6
DD02	7.0	6.5	4.5	0.80	97.4	94.9	97.4	96.6
DD03	7.0	6.5	4.0	0.85	95.4	95.4	99.0	96.6
DD04	6.5	5.5	4.5	0.80	96.5	94.6	97.8	96.3
DD05	7.0	6.0	4.0	0.85	94.4	95.4	99.0	96.2
DD06	6.5	6.0	4.0	0.85	94.3	95.3	99.0	96.2
DD07	6.0	5.5	4.5	0.80	95.6	94.6	98.1	96.1
DD08	7.0	6.5	3.5	0.90	92.5	95.7	99.4	95.8
DD09	7.0	5.5	4.0	0.85	93.8	94.9	98.8	95.8
DD10	6.0	5.0	4.5	0.80	95.6	94.1	97.7	95.8

### Sensitivity analysis

**Fig 3:**
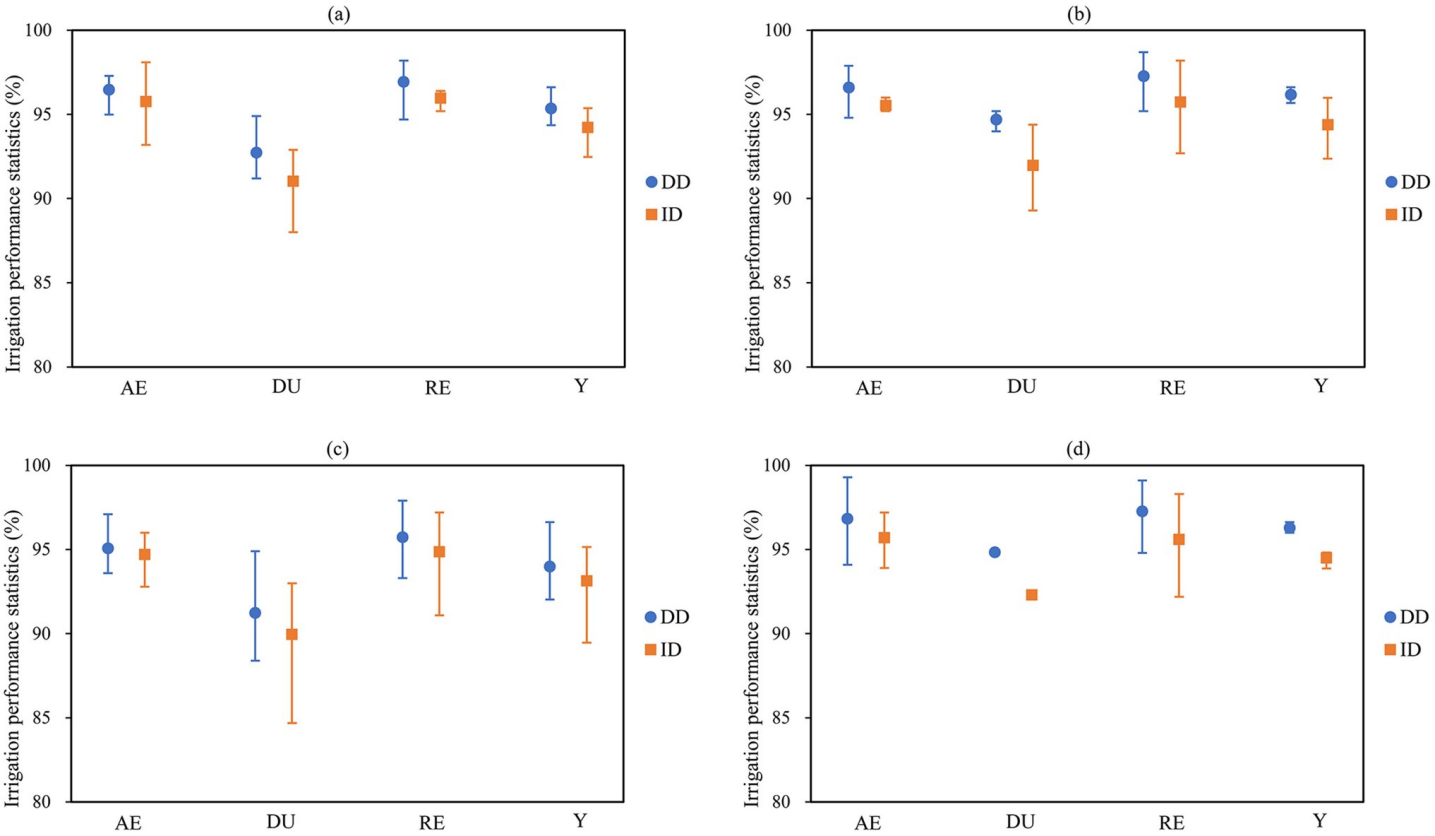
Sensitivity analysis of (a) inflow rate, (b) slope, (c) infiltration parameters, and (d) roughness coefficient. The vertical bars represent the deviations of irrigation performance indexes caused by the spatiotemporal variability of inflow rate, slope, infiltration parameters, and roughness coefficient.

The proposed optimal border irrigation scheme was analyzed over ranges of input parameters as follows: (*k*, *a*) from (6.4 mm·min^-α^, 0.68) to (7.2 mm·min^-α^, 0.71); *n* values of 0.10 ± 10%; *S* values of 0.0025 ± 20%; and *q* values of ± 10%. In the interest of brevity, the results of the analyses of sensitivity to the input parameters are reported without any adjustments to the optimal ID and DD irrigation schemes (ID01 and DD01 in Table 4). The AE, DU, RE and Y were still used as the performance indicators, and the irrigation performance deviations due to the spatiotemporal variability of the input parameters are shown in Fig 3. For the ±10% change in the inflow rate, the adverse impact on the DU was greater than that on the AE and RE (Fig 3a). The minimum DU values for the DD and ID schemes were 91.2% and 88.0%, respectively. The sensitivity of the slope was simulated over a range of slopes from 0.002 to 0.003. The irrigation performance of the DD scheme was excellent, and the AE, DU, RE and Y values of this varied-discharge irrigation method were almost greater than 95% (Fig 3b). The infiltration parameters (k, a) ranged from (6.4 mm·min^-α^, 0.68) to (7.2 mm·min^-α^, 0.71), and they had a greater impact on the irrigation performance than the other input parameters. In this case, the minimum AE, DU, RE and Y values were 93.6%, 88.4%, 93.3%, and 92.0% respectively for the DD scheme, and 92.8%, 84.7%, 91.1% and 89.5% respectively for the ID scheme (Fig 3c). For the ±10% change in the roughness coefficient, the irrigation performance (AE, DU, RE and Y) of the DD scheme was also better than that of the ID scheme (Fig 3d). Unlike the other input parameters, the roughness coefficient had little effect on the DU.

In general, the spatiotemporal variability in the input parameters decreased the irrigation performance of both the ID and the DD border irrigation schemes. The infiltration parameters had the greatest impact on the irrigation performance, followed by the inflow rate, slope and roughness coefficient. The result agreed with the research of Wang et al. [42] who simulated the contribution rate of the influence factors on continuous-discharge irrigation performance in closed-ended border irrigation systems and indicated that the most important influence factors were the inflow rate, infiltration coefficient (*k*) and infiltration index (*a*). When the input parameters changed, the DD scheme performed almost better than the ID scheme on all irrigation performance indexes. This partly explained the phenomenon that the DD border irrigation scheme achieved the most satisfactory irrigation performance in the field tests.

## Conclusions

The field testes and simulations indicated that the predesigned varied-discharge border irrigation scheme could improve the irrigation performance. For the irrigation event during the jointing stage in the North China Plain, the following suggestions were drawn from the study.

1. Decreased-discharge (DD) border irrigation has good irrigation performance and robustness against the spatiotemporal variability in the input parameters (infiltration parameters, roughness coefficient, slope and inflow rate), so this predesigned varied-discharge irrigation method is recommended.
2. The satisfactory irrigation performance was achieved when the ranges of the initial inflow rate, the inflow rate after the first change and the inflow rate after the second change were almost 6.5–7 L s^-1^m^-1^, 5.5–6.5 L s^-1^m^-1^ and 4–4.5 L s^-1^m^-1^, respectively. The appropriate cut-off distance ratio was closely related to the inflow rate after the second change, and the specific correspondence between them is as follows: 0.8 for 4.5 L s^-1^m^-1^, 0.85 for 4.0 L s^-1^m^-1^ and 0.9 for 3.5 L s^-1^m^-1^.
3. The sensitivity analysis showed that the infiltration parameters are the greatest influence factor on the irrigation performance. Therefore, more attention should be paid to the measurement of the infiltration parameters to design the optimal predesigned varied-discharge border irrigation scheme.

The predesigned varied-discharge border irrigation presented in this study was based on the irrigation event during the jointing stage in the North China Plain. The required application water depth and field characteristics may be greatly different for other irrigation events in other areas. Therefore, further research should be conducted in different fields to verify the universality of the proposed irrigation scheme.

## Supporting information

S1 File Observed relative elevations and calculated border slopes. (XLS)

S2 File Border geometry, Kostiakov parameters, roughness coefficient, inflow rate and irrigation performance of border irrigation experiments. (XLS)

S3 File Sensitivity analysis of inflow rate, slope, infiltration parameters, and roughness coefficient. (XLS)

S4 File Monthly precipitation and temperature from 2013 to 2018 during winter wheat growing seasons. (XLS)
